# Specifying sickle cell disease interventions: a study protocol of the Sickle Cell Disease Implementation Consortium (SCDIC)

**DOI:** 10.1186/s12913-018-3297-1

**Published:** 2018-06-27

**Authors:** Ana A. Baumann, Steven H. Belle, Aimee James, Allison A. King, the Sickle Cell Disease Implementation Consortium

**Affiliations:** 1 Brown School, Washington University in St. Louis, One Brookings Drive, Campus Box 1196, St. Louis, MO 63130 USA; 2 Graduate School of Public Health, University of Pittsburgh, Suite 605, 4420 Bayard St., Pittsburgh, PA 15260 USA; 3 Division of Public Health Sciences, 660 South Euclid Ave. Box 8100, St Louis, MO 63110 USA; 4 Program in Occupational Therapy; Department of Pediatrics, Division of Pediatric Hematology/Oncology; Division of Public Health Sciences, Department of Surgery, Washington University School of Medicine, St. Louis, MO 63108 USA

**Keywords:** Sickle cell disease, Implementation science, Scientific reproducibility, SCDIC

## Abstract

**Background:**

Sickle cell disease (SCD) is an inherited blood disorder that results in a lifetime of anemia, severe pain, and end-organ damage that can lead to premature mortality. While the SCD field has made major medical advances, much needs to be done to improve the quality of care for people with SCD. This study capitalizes on the Sickle Cell Disease Implementation Consortium (SCDIC), a consortium of eight academic sites aiming to test implementation strategies that could lead to more accelerated application of the NHLBI guidelines for treating SCD. This report documents the process to support the consortium by specifying the interventions being developed.

**Methods:**

This study consists of three steps. The Principal Investigator of each site and two site representatives who are knowledgeable of the intervention (e.g., study coordinator or the person delivering the intervention) will answer an online survey aiming to capture components of the interventions. This survey will be completed by the site representatives three times during the study: during the development of the interventions, after one year of the interventions being implemented, and at the end of this study (after 2 years). A site visit and semi-structured interview (Step 2) in the first year of the process will capture the context of the sites. Step 3 comprises of the development of a framework with the details of the multi-component SCDIC interventions at the sites.

**Discussion:**

The outcome of this study, a framework of the SCDIC, will enable accurate replication and extension of published research, facilitating the translation of SCD studies to diverse populations and settings and allowing for theory testing of the effects of the intervention components across studies in different contexts and for different populations.

**Trial registration:**

ClinicalTrial.Gov (#NCT03380351). Registered December 21, 2017.

## Background

Sickle cell disease (SCD) is an inherited blood disorder that results in a lifetime of anemia, severe pain, and end-organ damage that can lead to premature mortality [1]. SCD predominantly affects African Americans and other underrepresented minorities [2] and, while the field has made major medical advances [3], the average survival of African American individuals with SCD remains 30–45 years less than the average life expectancy for African Americans [4]. There are, however, efficacious interventions for SCD that can improve quality of life and life expectancy by reducing risks of stroke, pulmonary complications, need for transfusions, and reducing the number of pain episodes [5, 6]. Fortunately, the diagnosis of the disease is relatively simple, and mandatory newborn screening for SCD exists in all states so such interventions can be initiated early in the lifespan [7]. In 2014, the National Heart, Lung and Blood Institute released a set of evidence-based recommendations for treating SCD [8] but it is clear that the therapies proven to be efficacious are not reaching those in need [8, 9]. Implementation science, a field that aims to close the “quality gap” and support the transition of evidence-based intervention to usual care [10] can help improve the field of SCD care delivery [11].

Distinguishing between clinical studies and implementation studies may help clarify how implementation science can help improve the quality of care for people with SCD. Clinical studies aim to evaluate the efficacy or effectiveness of a specific intervention. Implementation studies, on the other hand, focus on *how* to successfully implement the intervention *in specific contexts* [12]. An implementation study aims to accelerate the adoption of an intervention or guideline by providers and/or systems of care, with the focus of the outcomes usually being at the provider and/or systems level [13]. In implementation studies, attention is given to the implementation strategies, defined as the processes by which evidence-based health innovations (e.g., audit and feedback, changes in the electronic record system, clinical supervision) are adopted and integrated into usual care [14]. These studies aim to change behaviors or settings at the organizational, practitioner, or patient level [15] with the goal of enhancing the adoption of an intervention or guideline. Implementation studies, therefore, require a paradigm shift [16] as they extend efficacy and effectiveness research focused on discovering *what* works to understanding *how* the implementation works *in specific contexts* [17].

The context of SCD care is complex and permeated with disparities. Treatment for SCD relies heavily on public insurance and healthcare programs [2], there are few specialized treatment centers [7], and the majority of people with SCD are minority and socioeconomically disadvantaged people who are also less likely than their counterparts to be linked to quality systems of care [18]. Children with SCD are at risk for life-threatening infections, strokes, acute chest syndrome, leg ulcers, pulmonary hypertension, among other complications [19]. Managing the disease involves multiple settings such as emergency department, as inpatient or outpatient, as well as at home and, for children, in school [20]. Proper management also includes both primary care and specialty care, though the latter may not always be available. Managing SCD is particularly complicated when adolescents are transitioning into adulthood, as they may have to move from a children’s clinic to adult clinics, adult emergency rooms, and hospitals. This may also be a time when individuals are taking on more responsibility for managing their own disease complications on their own, entering the workforce, and taking on other adult responsibilities [21].

### The sickle cell disease implementation consortium (SCDIC)

To address the challenges in managing SCD at this critical age juncture, the National Heart, Lung and Blood Institute (NHLBI), with co-sponsorship from the National Institute of Minority Health and Health Disparities, established a national research consortium to identify and test implementation strategies that could lead to more accelerated, consistent, and widespread application of the NHLBI guidelines. The Sickle Cell Disease Implementation Consortium (SCDIC): Using Implementation Science to Optimize Care of Adolescents and Adults with Sickle Cell Disease was established in 2016 [22] as a collaborative, multi-sector, research program that supports eight academic sites facilitated by a Data Coordinating Center – the Research Triangle Institute [11, 23]. The Consortium is charged with improving “the health and well-being of adolescents and adults with SCD in the US through the development of multi-modal, multi-sector interventions aimed at improving the rate at which patients with SCD receive routine primary care” [22].

Originally, each of the eight sites proposed a different intervention to achieve a common goal of improving the quality of care of people with SCD. To assist in cross-site collaboration, the SCDIC Executive Committee grouped site representatives from each of the eight academic sites to develop interventions in three main areas: (a) care redesign, (b) emergency department care, and (c) reducing the number of unaffiliated patients’ care. The specific research questions, designs and components of the interventions are currently being developed.

### Aims

The process described here was developed to support the sites in the SCDIC consortium by defining the components of the interventions with the goal of increasing the transparency and scientific reproducibility of the complex and multilevel studies being developed by the consortium sites.

### The importance of scientific reproducibility

One of the cornerstones of science is the ability to reproduce research findings [24]. In recent years, there has been a growing awareness of the need for transparency in studies to increase scientific reproducibility, including the launch of the clinicaltrials.gov in 2000, a registry where researchers record their methods and outcomes measures, and development of guidelines such as CONSORT [25], TIDier [26], and STaRI [27], to support transparency in research. Ideally these initiatives would facilitate replicability of major findings; however a number of studies have failed to replicate prominent findings or failed to achieve expected outcomes [28–31]. Insights from researchers reveal that, among other issues, the absence of clear descriptions of studies, including hypotheses for outcomes and descriptions of their interventions strategies [31, 32] make these studies virtually unusable, as they cannot be replicated, leading to inefficient use of research efforts and millions of dollars and efforts [33]. Unfortunately, the lack of detailed description of the interventions, including theoretical justification and review of the literature, is still prominent despite the push for publication guidelines [33, 34].

Describing components of complex interventions in the healthcare system, however, is challenging as often the relation between intervention, the implementation strategies and the contextual factors are dynamic, making the precise distinction among these components less clear [35]. A priori definition of the intervention components, the context, and the strategies used to implement the interventions can hopefully address many of the current problems in the intervention and implementation science fields: inconsistent labelling, disciplinary differences, vague descriptions (lack of transparency) and consequent difficult replication, and resultant inability to execute more precise meta-analyses [27, 35–40]. In other words, a standardized and detailed description of interventions allows accurate replication and extension of published research, facilitating the translation of studies to other populations and settings, allowing for theory testing of the effects of the intervention components across studies in different contexts and for different populations.

The description of a study needs to go beyond the correct and detailed description of methods and outcomes. A challenge to implementation research in any field is to capture what was proposed and what actually happened. There are at least two consequences of such mismatch and lack of transparency. First, by not adequately describing the study objective, methods, populations, interventions, outcomes measured and context, authors do not address, with high quality, their protocols in a proactive manner [33]. By proactively describing their studies in publications such as study protocols, researchers can contextualize their studies, allowing others to place their studies “into the context of totality of evidence” [41], and allowing others to reduce research waste by potentially doing similar studies when evidence has already been established. Second, it is well known that proposed studies differ from what actually happens when interventions are implemented [42, 43]. By clearly defining upfront the details of the interventions, and proactively defining what can be adapted, when and how adaptations can be made, it is hopefully easier to capture potential adaptations to the interventions once the study is ongoing [42]. In summary: “without accessible and usable report, research cannot help patients and their clinicians” [44].

To support the SCDIC, the process describe here aims to faciliatate the characterization of the interventions and strategies being developed by the SDIC in an effort to compare interventions. The primary outcome of this process will be a detailed framework of the SCDIC interventions that will support rigor and transparency, enable the Consortium to evaluate which intervention components were most effective in which settings, and will offer guidance to future efforts to improve treatment delivery and improve quality of life and life expectancy for adults living with SCD. This approach will also serve as a model for how other Consortia can describe, delineate, and evaluate interventions and implementation strategies across diverse sites.

## Methods

Guided by the recent report by the National Academy of Engineering (NAE) and the Institute of Medicine (IOM) calling for applying systems engineering methods to healthcare systems [45], we will focus on different levels that are interdependent and necessary to improve the quality of care. Accordingly, in the past years, approaches from the fields of engineering and business have been used to identify components of interventions [36, 46, 47] as predictors of outcomes [46, 48, 49]. This study is approved by the Washington University in St. Louis Human Research Protection Office (IRB Protocol # 201709005) and registered in the ClinicalTrial.Gov (#NCT03380351).

### Participants

Each site will have three representatives who will participate in this process: the PI of the study at that site and two people nominated by the PI as being the most knowledgeable of the intervention (e.g., study coordinator, interventionist/clinician). The interviews will be conducted in a half-day site visit. All data are being collected with the site representatives and there will be no contact with the patient participants of the SCDIC interventions. Because this study entails describing the interventions and how the interventions are delivered, the IRB has approved an information sheet to be provided to the site representatives instead of a written informed consent for the study.

### Data collection

The data collection for this study will have three phases: Step 1 will be a survey conducted prior to intervention implementation to capture the details of the proposed intervention. After one year, the survey will be conducted again, in addition to site visits (Step 2) to capture any differences between what was proposed and what is actually being implemented. Finally, Step 3 – which will take place on the second year 3 of this study, involves the sites’ representatives answering the survey again and developing a framework with detailed description of the SCDIC interventions.

#### Step 1: Developing the matrix

An online survey, adapted from a survey used in other consortia [50] will be developed to characterize the interventions. The survey, with multiple response options, will capture nine components of the interventions: (1) mode of delivery (e.g., face to face, video, phone), (2) materials used in the delivery of the intervention (e.g., manuals, pamphlets, videotapes), (3) where the intervention is being delivered (e.g., emergency department, primary care clinic, schools), (4) duration and intensity of the intervention (e.g., number of meetings, distribution of meetings over time), (5) scripting or how researchers or implementers plan to interact with providers and participants of the study (e.g., language provided to support interaction, general guidelines), (6) sensitivity to participant characteristics (e.g., visual supplements, oral supplements, literacy level); (7) interventionist characteristics (e.g., who is delivering the intervention, demographics of interventionist, how much training is required to deliver the intervention), (8) adaptability, or the extent to which an intervention can be modified (e.g., what, on what basis and when components can be modified), and (9) outcomes and hypothesis (i.e., planned outcomes being assessed, theoretical rationale for the outcomes, hypothesis being tested; when, and who will conduct the assessments).

To capture the implementation strategies, the survey will also incorporate recommendations on specifying and reporting implementation strategies [36] by asking the research teams to identify (a) who is delivering the intervention, (b) what are the actions, (c) who is the action target, (d) the temporality (i.e., when the strategy is being used), (e) the dose (i.e., how often the strategy is being used), (f) the implementation outcome likely affected by the strategy, and (g) the theoretical justification for using the strategy.

#### Step 2: Site visits and follow up calls

Because we know that the interventions proposed will probably be adapted during the implementation process [42], we will ask the site representatives to answer the survey one year after the interventions are being implemented. Additionally, the study team will do a half-day site visit to each of the sites to learn more about the context of each study. If needed, follow up calls will be done to support the completion of the matrix.

#### Step 3: Refining and agreeing with the framework

At the end of the study, representatives of the sites will answer the survey again. The framework, which will be based on the three data points, and will contain what was proposed compared to what was actually delivered, will be presented to all sites in a webinar meeting during which feedback will be gathered regarding accuracy of the data.

### Data analysis

Simple descriptive, data comparative, and correlational statistics will be used to analyze data captured via the survey. The interviews will be transcribed and data analysis will be based upon principles from grounded theory using emergent coding strategies [51–55]. Coding will begin with open coding of the transcripts to develop topics and codes, and then a codebook will be developed that will list codes, code definitions, and sample code data. The codebook will be tested and iteratively revised until coder agreement is high and no modifications to the codebook is deemed necessary. At least two raters (the PI and a research assistant) will code each transcript using the codebook. Code queries will be used to examine all data under each code, which will be reviewed and analyzed to determine common meanings and themes. We will evaluate thematic saturation (i.e., no new themes will be captured with further analysis) and follow the Consolidated criteria for reporting qualitative research (COREQ) guidelines when reporting our findings [56].

## Discussion

This study is timely and important. First, this is a unique opportunity to support a consortium of eight academic sites aiming to improve the quality of life of adolescents and adults with SCD. By doing it prospectively instead of retrospectively, as has been done with other consortia [47, 50, 57, 58], one can compile the data and report intervention characteristics as they are being developed and implemented. This proactive approach should facilitate identifying and helping to address differences in labelling by the different stakeholders in the consortium (e.g., the word “implementation” is often used with different meanings by hematologists and implementation scientists) and increase the transparency in descriptions of the interventions. Additionally, by asking site representatives to describe the study objective, methods, populations, interventions, and outcomes in “the context of totality of evidence” [41], we hope to reduce research waste and push the field of SCD further.

By clearly detailing the interventions and planning for potential adaptations prospectively, and surveying site representatives at the end of the studies, the study described here will contribute to the fields of SCD and implementation science as we will be able to capture the differences in what was proposed and what was actually delivered. It is our hope that this process will help us delineate methodologies to capture the adaptations made to interventions being implemented in different settings (e.g., hospitals, schools) as part of this rich consortium.

In summary, the framework of the SCDIC will provide accurate replication and extension of published research, facilitating the translation of studies to diverse populations and settings, allowing for theory testing of the effects of the intervention components across studies in different contexts and for different populations [46, 57, 58].

## Abbreviations

SCD: Sickle cell disease
SCDIC: Sickle cell disease consortium
